# Clinical utility of genetic testing in Indian children with kidney diseases

**DOI:** 10.1186/s12882-023-03240-z

**Published:** 2023-07-18

**Authors:** Anshuman Saha, Shahenaz F. Kapadia, Kinnari B Vala, Himanshu V. Patel

**Affiliations:** 1 Department of Pediatric Nephrology, Institute of Kidney Diseases and Research Centre, Gujarat University of Transplantation Sciences, Ahmedabad, India; 2 Department of Nephrology, Institute of Kidney Diseases and Research Centre, Gujarat University of Transplantation Sciences, Ahmedabad, India

**Keywords:** Massively Parallel Sequencing, Genetic Testing, Children

## Abstract

**Background:**

Kidney diseases with genetic etiology in children present with an overlapping spectrum of manifestations. We aimed to analyze the clinical utility of genetic testing in the diagnosis and management of suspected genetic kidney diseases in children.

**Methods:**

In this retrospective study, children ≤ 18 years in whom a genetic test was ordered were included. Clinical indications for genetic testing were categorized as Glomerular diseases, nephrolithiasis and/or nephrocalcinoses, tubulopathies, cystic kidney diseases, congenital abnormality of kidney and urinary tract, chronic kidney disease of unknown aetiology and others. Clinical exome sequencing was the test of choice. Other genetic tests ordered were sanger sequencing, gene panel, multiplex ligation-dependent probe amplification and karyotyping. The pathogenicity of the genetic variant was interpreted as per the American College of Medical Genetics classification.

**Results:**

A total of 86 samples were sent for genetic testing from 76 index children, 8 parents and 2 fetuses. A total of 74 variants were reported in 47 genes. Out of 74 variants, 42 were missense, 9 nonsense, 12 frameshifts, 1 indel, 5 affected the splicing regions and 5 were copy number variants. Thirty-two were homozygous, 36 heterozygous and 6 were hemizygous variants. Twenty-four children (31.6%) had pathogenic and 11 (14.5%) had likely pathogenic variants. Twenty-four children (31.6%) had variants of uncertain significance. No variants were reported in 17 children (22.3%). A genetic diagnosis was made in 35 children with an overall yield of 46%. The diagnostic yield was 29.4% for glomerular diseases, 53.8% for tubular disorders, 81% for nephrolithiasis and/or nephrocalcinoses, 60% for cystic kidney diseases and 50% for chronic kidney disease of unknown etiology. Genetic testing made a new diagnosis or changed the diagnosis in 15 children (19.7%).

**Conclusion:**

Nearly half (46%) of the children tested for a genetic disease had a genetic diagnosis. Genetic testing confirmed the clinical diagnoses, changed the clinical diagnoses or made a new diagnosis which helped in personalized management.

## Introduction

With rapid advances in gene sequencing technology, an increasing number of Mendelian forms of kidney diseases are being identified in children with steroid-resistant nephrotic syndrome (SRNS), tubulopathy, nephrolithiasis and/or nephrocalcinosis, cystic kidney disease and hemolytic uremic syndrome. In some kidney diseases like congenital anomalies of the kidney and urinary tract (CAKUT), genetic etiology is not completely characterized. Many of the children with these kidney diseases progress to chronic kidney disease (CKD) [1, 2]. Nearly 30% of children and 5–30% of adults with CKD have one of the 450 genes mutated to explain the etiology of CKD [3]. Identifying monogenic causes has significant benefits in the management of children with kidney diseases. It ends the diagnostic journey by unequivocally establishing a diagnosis, allows informed treatment and helps in detecting and monitoring extra-renal complications. It also helps in detecting the disease in mildly symptomatic or asymptomatic family members by extended family screening. Knowledge of inheritance patterns and specific genetic variants guides further reproduction decisions and detection of the genetic variant in the fetus.

There is a significant shift in the paradigm of genetic testing in pediatric nephrology clinical practice with a move from a rare specialized and expensive test to the emergence as one of the common diagnostic methods used in the clinical setting. This is mainly due to the rapid progress in massively parallel sequencing and decreasing cost of massively parallel sequencing technology [4]. Massively parallel sequencing has also allowed the identification of new disease-causing genes and helped in understanding the molecular pathophysiology of many inherited diseases of childhood [3]. However, clear information regarding the likely outcome and impact of genetic testing is still missing. While the data on the clinical utility of genetic testing in specific disease groups in Indian children are available, information about its impact on diagnosis and management in an unselected group of children with kidney diseases is limited. We report our experience about the clinical utility of genetic testing in children with kidney diseases.

## Methods

### Study setting

The study was done at a tertiary care public nephrology teaching institute in India.

### Ethical approval

The study was approved by institutional ethics committee registered at CDSCO with registration no ECR/143/Inst/GJ/2013/RR-19, reference letter: IKDRC-ITS EC/App/31Jul20/2. Informed consent was obtained from all subjects and/or their legal guardian(s).

### Study design and Participants

This was a retrospective study where medical records of all children under the age of 18 years treated at a tertiary care nephrology center in whom a genetic etiology was suspected, and a genetic test was ordered between September 2016 to January 2021 were analyzed. Demographic details, history of consanguinity in parents, family history of similar illness, extrarenal and syndromic features were noted.

### Disease categories

kidney diseases were categorized under the following disease categories: glomerular disease, nephrolithiasis and/or nephrocalcinosis, tubulopathy, cystic kidney disease, CKD with unknown etiology (CKDu), congenital abnormality of kidney and urinary tract (CAKUT) and others.

### Genetic testing

The genetic testing method (karyotyping, multiplex ligation-dependent probe amplification (MLPA), Sanger sequencing, gene panel and clinical exome sequencing (CES)) was noted. The test results were interpreted as per the American College of Medical Genetics (ACMG) classification [5]. Where possible, family segregation studies were performed.

A genetic test was ordered by sending 5 ml blood in EDTA to a commercial laboratory.CES or targeted nephrotic syndrome panel were the investigations of choice when a genetic cause was suspected. Selective capture and sequencing of the protein-coding regions of the genome/genes were performed with a custom capture kit using the DNA extracted from blood. The libraries were sequenced to mean > 80-100X coverage on the Illumina sequencing platform (Illumina Inc., CA, USA). Genome Analysis Tool Kit best practices framework was followed for the identification of variants in the sample using Sentieon (v201808.01) [6]. Gene annotation of the variants was performed using the VEP program [7] against the Ensemble release 91 human gene model [8]. In addition to SNVs and small Indels, copy number variants (CNVs) were screened from sequence data using the ExomeDepth (v1.1.10) method [9]. Clinically relevant variants were annotated using published variants in literature and a set of diseases databases—ClinVar, OMIM, GWAS, HGMD (v2018.3) and SwissVar. Common variants were filtered based on allele frequency in 1000Genome Phase 3, ExAC (v1.0), gnomAD (v2.1), EVS, dbSNP (v151), 1000 Japanese Genome and internal Indian population database. The non-synonymous variant's effect was evaluated using multiple algorithms such as PolyPhen-2, SIFT, VariantTaster2 and LRT. Only non-synonymous and splice site variants found in the clinical exome panel consisting of 8332 genes were used for clinical interpretation. Silent variations that do not result in any change in amino acid in the coding region were not reported.

**Fig. 1:**
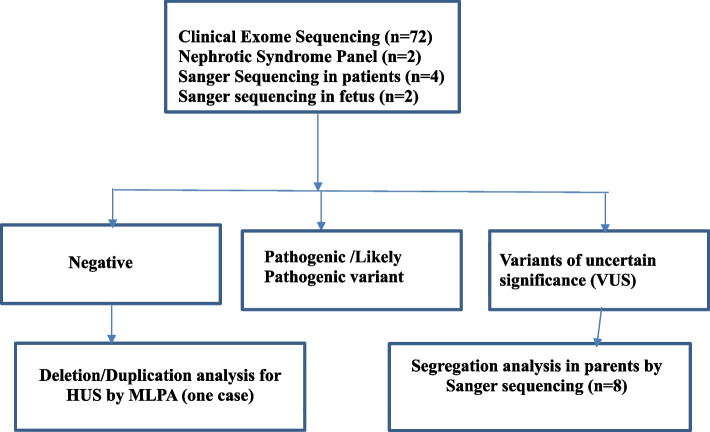
Flow Diagram to indicate the protocol followed for testing of samples

Sanger sequencing was done for some of the cases in parents of a proband when there was a strong suspicion of a particular gene, but more commonly to confirm the variant in the case of a variant of uncertain significance (VUS). MLPA was ordered for one of the selected case when there was no variant found in CES but when a copy number variation (CNV) was strongly suspected like in HUS. The SALSA MLPA Probemix P236 CFH Region from MRC Holland was used for the detection of deletions or duplications in the CFH, CFHR1, CFHR2, CFHR3, CFHR4 and CFHR5 genes in genomic DNA isolated from human peripheral whole blood specimens. Copy number differences of various exons between test and control DNA samples were detected by analyzing the MLPA peak patterns. Segregation analysis could not be done in all cases due to limited funds. The work flow diagram has been depicted in Fig. 1. The clinical impact of genetic testing was assessed by determining its utility in confirming the clinical diagnosis, making a new diagnosis, reclassifying a disease, reverse phenotyping, changing treatment and genetic counselling.

CES was done in 72 samples, targeted Nephrotic Syndrome gene panel in 2 (genes covered in Nephrotic Syndrome gene panels are given in Table 1), Sanger sequencing in 14 and MLPA in 1. Of the 14 Sanger sequencing performed, 2 were done to confirm CES variants, 8 in parents of children to confirm inheritance pattern and segregation, 2 for screening known genes in familial context or established syndrome, and 2 in the fetus. Karyotyping was done in 3 children.

**Table 1:**
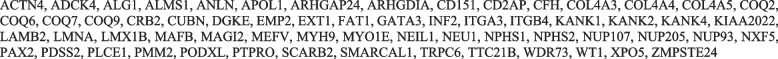
Genes covered in nephrotic syndrome gene panel

### Statistical analysis

Descriptive statistics were used for the study. The yield of genetic testing for various disease categories were expressed as percentage. The categorical variables were described as median. The numbers were small for any comparative statistical analysis.

## Results

### Patient characteristics

Genetic testing was performed in 86 individuals between September 2016 to January 2021, consisting of 76 index children, 8 parents, and 2 fetuses. Overall, the median age (IQR) of children at the onset of disease was 48 months (12–96 months). Forty out of 76 children (52.6%) were males. Nine families (10.5%) had a history of consanguinity. A family history of the same disease was present in only 4 families. The clinical disease categories are provided in Table 2. Glomerular diseases (44.7%) were the most common disease category for which a genetic test was ordered, in which the majority were steroid-resistant nephrotic syndrome (32.8%). Notably, 5.2% of the patients presented with kidney failure of unknown origin.

**Table 2:** Clinical characteristics and disease category of patients

Age in months (median, IQR)	72 (24–120)
Age in months at disease onset (median, IQR)	48 (12–96)
Sex Male	40 (52.6%)
Consanguinity	7 (10.5%)
Glomerular diseases SRNS Atypical HUS Dense deposit disease + HUS C3 GN Alport Syndrome	34 (44.7%) 25 04 01 01 03
Tubular disorders Fanconi syndrome Distal RTA Bartter syndrome Rickets Low molecular weight proteinuria Unclassified	13 (17.1%) 05 02 02 01 01 02
Nephrolithiasis/calcinosis Primary hyperoxaluria Nephrolithiasis with hyperuricemia FHHNC	11 (14.4%) 08 02 01
Cystic kidney disease Nephronophthisis related ciliopathy Bardet Biedl Syndrome Glomerulocystic kidney disease ADPKD ARPKD	10 (13.1%) 05 02 01 01 01
CKD of unknown aetiology	04 (5.2%)
CAKUT	02 (2.6%)
Others	02 (2.6%)
Total	76 (100%)

*SRNS* Steroid Resistant Nephrotic Syndrome, *HUS* Hemolytic Uremic Syndrome, *C3 GN* C3 Glomerulonephritis, *RTA* Renal Tubular Acidosis, *FHHNC* Familial Hypomagnesemia Hypercalciuria And Nephrocalcinosis, *ADPKD* Autosomal Dominant Polycystic Kidney Disease, *ARPKD* Autosomal Recessive Polycystic Kidney Disease, *CKD* Chronic Kidney Disease

### Variant identification and diagnostic yield

A total of 74 variants in 47 genes were reported in 59 out of 76 index children (77.6%). Seventeen children (22.3%) did not have any genetic variant. Out of all 74 variants 51 were novel and were not previously reported. Eighteen out of 51 novel variants were pathogenic/likely pathogenic and 33 were VUS. Forty-two variants were missense, 9 nonsense, 12 frameshifts, 1 indel, 5 variants affected the splicing regions and 5 were copy number variants (CNV).

A genetic diagnosis was made in 35 children (overall yield 46%; 35/76). Twenty-four children (31.5%) had pathogenic and 11(14.4%) had likely pathogenic variants. By disease category, the diagnostic yield was 29.4% in glomerular diseases (10 of 34 children), 53.8% in tubular disorders (7 of 13 children), 81% in nephrolithiasis and/or nephrocalcinosis (9 of 11 children), 60% in ciliopathies/cystic kidney diseases (6 of 10 children) and in 50% of CKD of unknown etiology (2 of 4 children) (Table 3). Of the 17 distinct monogenetic disorders detected, primary hyperoxaluria (*n* = 6) followed by Alport syndrome (*n* = 5) were the most common genetic diagnoses in the cohort. In 15 children (19.7%) genetic testing provided a new diagnosis or changed the clinical diagnosis. It revised the clinical diagnosis in 4 children. Clinical characteristics and details of genetic variants of children diagnosed with a genetic disease are depicted in Table 4. Twenty-four out of 76 children (31.5%) had variants of uncertain significance (VUS). Six children with pathogenic variants had also additional VUS. The details of children with VUS and children without a genetic variant are depicted in Tables 5 and 6 respectively.

**Table 3:** Outcome of genetic testing in patients

Disease Category	**Glomerular Diseases**	**Tubular Disorders**	**Nephrolithiasis/Nephrocalcinosis**	**Cystic Kidney Diseases**	**CKD of unknown aetiology**	**Congenital Abnormality of Kidney and Urinary Tract**	**Others**
Positive Diagnosis/yield n (%)	10 of 34 (29.4%)	7 of 13 (53.8%)	9 of 11 (81%)	6 of 10 (60%)	2 of 4 (50%)	0 of 2 (0%)	1 of 2 (50%)
New Diagnosis n (%)	3 (8.8%)	2 (15.3%)	5 (45.4%)	3 (30%)	2 (50%)	0 (0%)	1 (50%)

**Table 4:** Clinical and genetic details of children with a positive genetic diagnosis

Case	Age mo	Sex	Family history of similar illness	Disease category	Clinical diagnosis	Gene	Location	Variant	Zygosity	ACMG Classification	Genetic diagnosis (AD/AR/XL)	Utility of genetic testing
1	24	F	No	5	Bardet Biedl Syndrome	CEP164	Exon 23	c.2863G > T p.Glu955Ter	Homo	Pathogenic	Nephronopthisis 15 (AR)	New diagnosis
2	5	F	No	3	Primary Hyperoxaluria	AGXT	exon2	c.245G > A p.Gly82Glu	Homo	Pathogenic	PH-1 (AR)	Confirmed the diagnosis and diagnosed the exact type of PH
4	10	M	No	3	Primary Hyperoxaluria	AGXT	Exon1	c.32C > G, c.107G > A p.Pro11Arg, p.Arg36His	Homo Homo	Pathogenic	PH-1 (AR)	Confirmed the diagnosis and diagnosed exact type of PH, Fetus in subsequent pregnancy aborted after the same mutation detected in the fetus
5	96	M	No	2	FSGS rapidly progressed to ESRD, Pre-transplant evaluation for disease recurrence	NPHP1	Exon 1–20	Deletion of the region	Homo	Likely pathogenic	Nephronophthisis 1 (AR)	New diagnosis, changed the diagnosis from FSGS to ciliopathy, clarified disease recurrence risk post-transplant
7	4	F	No	1	RTA	ATP6V0A4	Exon 13	c.1185del p.Tyr396ThrfsTer12	Homo	Pathogenic	d RTA with preserved hearing (AR)	Confirmed the diagnosis and led to finding other features like SNHL
12	10	M	No	3	Hyperuricemic nephrolithiasis	HPRT1	Exon 3	c.212dupG p.Tyr72LeufsTer2	Hemi	Pathogenic	Lesch nyhan syndrome (XLR)	New diagnosis
13	10	F	No	1	RTA	SLC4A1	Exon 19	c.2573C > A p.Ala858Asp	Homo	pathogenic	d RTA (AR)	Confirmed the diagnosis
14	1	M	No	3	Uric acid stone, Hyperuricemia	HPRT1	intron 1	c.27 + 2 T > G 5 slice error	Hemi	Pathogenic	Lesch nyhan syndrome (XLR)	Diagnostic, new diagnosis, second issue fetus same mutation, aborted
16	120	F	No	6	Nephronopthisis	NPHP1	Deletion probable	chr2:g.(?-109,974,999)-110,213,122-?del	Homo	Likely Pathogenic	Nephronopthisis1, Joubert, SLS (AR)	Diagnostic and confirmatory
21	1	M	No	1	Bartter syndrome	SLC12A1	Exon22	c.2716C > T p.Gln906Ter	Homo	Pathogenic	bartter syndrome 1 (AR)	Diagnostic and confirmatory
22	4	M	No	3	Primary Hyperoxaluria	AGXT	Exon2	c.245G > A p.Gly82Glu	Homo	Pathogenic	PH-1 (AR)	Confirmatory and disease classification
24	22	F	No	2	Primary SRNS, FSGS	LAMB2	Exon29, 9	c.4882dup, c.1045 T > A p.Ala1628GlyfsTer4, p.Cys349Ser	Compound Hetero	Pathogenic	Pierson syndrome (AR)	New diagnosis
27	12	F	No	3	Nephrolithiasis	HOGA1	Exon1	c.134C > T p.Pro45Leu	Homo	Likely pathogenic	PH-3 (AR)	New diagnosis and disease classification
29	24	M	Yes	2	Primary SRNS	PLCE1	Exon 20	c.4848del p.Gln1616HisfsTer12	Homo	Pathogenic	DMS NS 3 (AR)	Diagnostic
30	47	F	No	1	Proximal RTA	FAH	Exon3	c.192G > T p.Gln64His	homo	Pathogenic	Tyrosinemia type 1 (AR)	New diagnosis
31	6	F	No	3	Primary Hyperoxaluria	AGXT	Exon 2	c.302 T > C p.Leu101Pro	Homo	Pathogenic	PH-1 (AR)	Confirmatory and disease classification
32	96	F	No	5	Nephronopthisis	DYNC2H1	Exon 65, Intron 30	c.9844C > T, c.4612-4A > G p.Arg3282Ter	Compound Hetero	Pathogenic	Short Rib Thoracic Dysplasia 3 with or without polydactyly	New diagnosis
34	1	M	No	1	Nephropathic cystinosis	CTNS1	Exon7	c.461G > T p.Ser154Ile	Homo	Pathogenic	Nephropathic cystinosis	Confirmatory
35	105	F	No	6	CKD of unknown aetiology	COL4A4	Exon 47	c.4717del p.Ala1573ProfsTer30	Homo	Pathogenic	Alport syndrome (AR)	New diagnosis. Led to reverse phenotyping and detecting the same variation in the younger brother who had hematuria
37	24	M	No	2	Frasier/Denys Drash Syndrome	WT1	Exon 4	c.896C > T Pp.Ser299Phe	Hetero	Likely pathogenic	Denys Drash Syndrome (AD)	Diagnostic, got a genetic diagnosis after 12 years
38	96	F	No	8	Primary hypoparathyroidism	AIRE	Exon2, 2	c.165del, c.195G > T p.Gln57ArgfsTer11,p.Trp65Cys	Compound Hetero	Pathogenic	Autoimmune PES type 1 (AR)	New diagnosis
42	24	F	No	1	Hypophosphatemic rickets	PHEX	Exon15	c.1645C > T p.Arg549Ter	Hetero	Pathogenic	XLHR	Confirmatory
44	120	M	No	6	CKD of unknown etiology	COL4A5	Exon 15	c.884G > A p.Gly295Asp	Hemizygous	Pathogenic	XLAS	New diagnosis
48	4	M	No	1	Fanconi syndrome	SLCA2	Exon3	c.339del p.Phe114LeufsTer16	Homo	Pathogenic	Fanconi Bickel Syndrome (AR)	New diagnosis, Diagnostic and avoided liver biopsy
49	0	M	No	5	ARPKD	PKHD1	Exon 54,65	c.8501dup,c.11542G > C p.Val2836SerfsTer4,p.Val3848Leu	Compound Hetero	Pathogenic	ARPKD	confirmatory
51	60	M	No	2	Primary SRNS, FSGS	COL4A3	Exon 3	c.172_178dup p.Pro60ArgfsTer12	Homo	Pathogenic	FSGS COL4A3 (AR)	Diagnostic, new diagnosis
57	120	M	Yes	9	Alport syndrome	COL4A5	Exon 44	c.3850G > T p.Gly1284Ter	Hemi	Pathogenic	XLAS	Confirmatory
59	120	M	No	5	Nephronopthisis SLS	SDCCAG8	Exon 16	c.1885dup p.Arg629LysfsTer5	Homo	Pathogenic	SLS (AR)	Diagnostic, stopped immunosuppression which was previously started thinking chronic glomerulonephritis
63	16	F	No	2	Primary SRNS	NPHS2	Exon 3	c.412C > T p.Arg138Ter	Homo	Pathogenic	FSGS Podocin mutation (AR)	Diagnostic
66	119	F	No	9	Alport Syndrome	COL4A3	Exon 22	c.1343delC p.Pro448LeufsTer10	Homo	Pathogenic	ARAS	Confirmatory
67	48	F	No	2	Primary SRNS/FSGS	WT1	intron9	c.1432 + 5G > A(5' proximal site) p.Pro739Ala	Hetero	Pathogenic	Frasier syndrome (AD)	New diagnosis. Reverse phenotyping revealed complete sex reversal on karyotyping
70	132	F	No	3	Primary Hyperoxaluria	KCNJ1	Exon2	c.658C > T p.Leu220Phe	Homo	Likely Pathogenic	Bartter syndrome type 2 (AR)	New diagnosis. Reverse phenotyping indeed revealed Bartter syndrome on investigation
71	170	M	No	3	Primary Hyperoxaluria	AGXT	Exon 1,5	c.33dupC, c.577dupC p.Lys12GlnfsTer156,p.Leu193ProfsTer32	Compound Hetero	Pathogenic	PH-1 (AR)	Confirmatory
72	192	F	No	5	ADPKD	PKHD1	Exon3, Intron 5	c.4870C > T C.390 + 5G > T p.Arg1624Trp 5 slice site	Compound Hetero	Likely pathogenic	ARPKD	Changed the diagnosis
76	60	F	No	9	Alport syndrome	COL4A4	Exon 47	c.4717del; p.Ala1573ProfsTer30	Homo	Pathogenic	ARAS	Confirmatory

Disease category: 1. Tubular diseases 2. Steroid resistant nephrotic syndrome (SRNS) 3. Nephrolithiasis and/or Nephrocalcinosis 4. Congenital anomalies of kidney and urinary tract 5. Cystic Kidney Diseases 6. Chronic kidney disease (CKD) of unknown aetiology 7. Hemolytic uremic syndrome (HUS) 8. Others 9. Alport Syndrome

**Table 5:** Clinical and genetic details of children who had VUS

Case	Age in mo	Sex	Family History	Disease Category	Clinical diagnosis	Gene	Location	Variant	Zygosity
3	60	M	Nil	7	C3 Glomerulopathy	WT1	Exon 9	c.749G > A(p.Arg250Gln)	Heterozygous
6	9	F	Nil	5	Glomerulocystic kidney disease	PKD2		c.103_104delinsAA(p.Al135Asn)	Heterozygous
11	132	F	Nil	2	FSGS	PLCE1	Exon 10	c.6655 T > A(p.Phe2219Ile)	Heterozygous
17	12	M	Nil	2	SRNS	NUP133	Exon 10	c.1196C > T(p.Ser399Phe)	Heterozygous
19	72	M	Nil	2	SRNS	TRPC6	Exon 2	c.299A > C(p.Glu100Ala)	Homozygous
25	24	F	Nil	7	Atypical HUS	SPTB	Exon 26	c.5617G > A(p.Ala1873Thr)	Heterozygous
26	96	F	Nil	5	Nephronopthisis related ciliopathy	PKD1	Exon 15	c.6593C > T(p.Pro2198Leu)	Heterozygous
28	12	M	Nil	2	SRNS	NUP93	Exon 13	c.1463A > G(p.His488Arg)	Homozygous
40	132	F	Nil	7	Atypical HUS and Dense deposit Disease	CFB	Exon 23	c.2990G > A(p.Trp997Ter)	Heterozygous
43	120	F	Nil	5	Bardet Biedl Syndrome	PKD1	Exon 25	c.9113C > G(p.Pro3038Arg)	Heterozygous
46	96	M	Nil	2	SRNS	NPHP4	Exon 22 Exon 17	c.3175G > A(p.Ala1059Thr) c.2251G > A(p.Val751Ile)	Heterozygous
47	48	M	Nil	2	SRNS	INF1	Exon 19	c.2848C > T(p.Arg950Trp)	Heterozygous
52	60	M	Nil	7	Antifactor H antibody positive HUS	CFHR1,3	Deletion probable		
56	84	F	Nil	2	SRNS	FAT1, DGKE, FAT1	Exon10,11,25	c.7730 T > C(p.Val2577Ala) c.1442G > C(p.Gly481Ala) c.3850G > T(p.Asp218Gly)	Heterozygous
60	120	F	Nil	1	Renal Rickets	FAT1, EYA1	Exon19,Exon10 Intron14	c.10622A > G(p.Tyr3541Cys) c.5488C > T(p.His1830Tyr) c.1360 + 2C > T	
64	192	F	Nil	6	CKD	BBS4	Exon 11	c.760G > A(p.Val254Ile)	Homozygous
65	156	M	Nil	2	SRNS	NUP 205	Exon 7	c.938G > A(p.Arg313His)	Heterozygous
68	24	F	Nil	2	SRNS	INF2	Exon 8	c.1049C > T(p.Pro350Leu)	Heterozygous
69	60	F	Nil	1	Tubulopathy	ADCY10	Exon 19	c.2414 T > A	Heterozygous
75	72	M	Nil	7	Antifactor H antibody positive HUS	CFHR3 CFHR1 STIM1 COL4A5	Exon1,2,3,6, intron4 Exon 3,5,6 Exon 6 Exon 19	c.692A > g(pTyr231Cys) c.1095_1103del (p. Leu366_Gly368del)	Heterozygous Hemizygous
15	12	M	Nil	1	Bartter Syndrome	SLC12A1	Exon 14	c.1685C > T(p.Ala562Val)	Homozygous
53	180	M	Nil	3	Primary Hyperoxaluria	GRHPR	Exon 4	c.349 T > C(p.Ser117Pro	Homozygous
62	24	F	yes	3	Familial Hypomagenesemia Hypercalciuria and Nephrocalcinosis	CLDN16	Exon2	c.374 T > C(p.Phe125Ser)	Homozygous
73	70	M	Nil	7	Antifactor H antibody positive HUS	CFHR1,3	Chr1:g del	Chr1:g Del	Homozygous

Disease category: 1. Tubular diseases 2. Steroid resistant nephrotic syndrome (SRNS) 3. Nephrolithiasis and/or Nephrocalcinosis 4. Congenital anomalies of kidney and urinary tract 5. Cystic Kidney Diseases 6. Chronic kidney disease (CKD) of unknown aetiology 7. Hemolytic uremic syndrome (HUS) 8. Others 9. Alport Syndrome

**Table 6:** Clinical details of children with no genetic variant

Case	Age in mo	Sex	Family History	Disease Category	Clinical Diagnosis
9	60	M	Nil	1	Tubulopathy
37	108	M	Nil	1	Proximal RTA
61	84	M	Nil	1	Dent Disease
18	84	M	Nil	2	SRNS/FSGS
8	60	F	Nil	2	SRNS/FSGS
23	60	F	Nil	2	SRNS/FSGS
33	48	M	Nil	2	SRNS/FSGS
39	84	M	Nil	2	SRNS/FSGS
45	48	M	Nil	2	SRNS/FSGS
50	24	M	Nil	2	SRNS/FSGS
58	24	M	Nil	2	SRNS/FSGS
74	12	M	Nil	2	SRNS/FSGS
20	192	M	Nil	4	Syndromic CAKUT
10	132	M	Yes, younger brother had similar complain of CKD and retinitis pigmentosa	5	Senior Loken Syndrome
55	168	F	Nil	6	CKD
54	132	F	Nil	6	CKD
41	48	M	Nil	8	Idiopathic infantile calcinosis

Disease category: 1. Tubular diseases 2. Steroid resistant nephrotic syndrome 3. Nephrolithiasis and/or Nephrocalcinosis 4. Congenital anomalies of kidney and urinary tract 5. Cystic Kidney Diseases 6. Chronic kidney disease of unknown aetiology 7. Hemolytic uremic syndrome 8. Others 9. Alport Syndrome

The median age of the children with a genetic disease at disease onset was 24 months (range 10–108 months) compared to 48 months (range 48–82 months) in children without a genetic diagnosis. The majority, 28 (80%) had an autosomal recessive inheritance and 6 (17.1%) had a history of consanguinity. Syndromic features were noted in 6 children (17.1%).

### Glomerular diseases

Among glomerular diseases, steroid-resistant nephrotic syndrome (SRNS) was the most common indication for ordering a genetic test (*n* = 25; 32.8%). All were initial SRNS. Seven children (28%) had a pathogenic variant, 9 (36%) had VUS while 9 children did not have any variant. Four of the seven (57%) with a pathogenic variant had the age of onset less than 24 months. Two children had a pathogenic variant in *WT1* and 1 each in *NPHS2, PLCE1, COL4A3, LAMB2* and *NPHP1*. One child with WT1 had clinical phenotype of Frasier vs Denys Drash syndrome (Case 37) and other with WT1 (Case 67) had SRNS without any other syndromic feature. Genetic testing resulted in a new diagnosis in 3 children (12%) with a clinical diagnosis of SRNS [nephronophthisis-1 (case 5), Pierson syndrome (case 24) and Frasier syndrome (case 67)]. In case 5 the patient was clinically diagnosed as FSGS as he had nephrotic syndrome at presentation along with kidney failure and biopsy had revealed sclerosed glomeruli. CES revealed nephronophthisis which gave the genetic diagnosis and predicted post kidney transplantation recurrence. In the other two children it helped in reverse phenotyping. In case 67 who was phenotypically female, karyotyping was done after CES which revealed 46 XY suggesting complete sex reversal. In case 24 with Pierson syndrome however there was no microcornea. In 5 children, immunosuppression was discontinued following genetic diagnosis. Nine children with VUS also had very severe disease resistant to both prednisolone and calcineurin inhibitors, similar to those with an identified genetic cause. Causality could not be established in those with VUS as genetic tests in parents were inconclusive and functional analysis could not be done. Four children with a pathogenic variant and 5 with VUS progressed to end-stage kidney disease (ESKD). In contrast, only 1 child without any genetic variant progressed to ESKD.

CES was done in all children with alternative complement pathway abnormality (atypical HUS *n* = 4, C3GN *n* = 2); however, MLPA was done in only 1 child due to cost constraints. VUS were identified in all children in *WT1, SPTB, CFB, CFHR1* and *CFHR3* genes. In 3 children with suspected Alport syndrome, diagnosis was confirmed in all (1 X linked Alport Syndrome and 2 Autosomal recessive Alport Syndrome).

### Cystic kidney diseases

Ten children with suspected cystic kidney disease were evaluated for a genetic variant. Five had suspected nephronophthisis, 2 were suspected Bardet Biedl Syndrome, while 1 patient each was clinically diagnosed with glomerular cystic kidney disease, autosomal dominant polycystic kidney disease (ADPKD), autosomal recessive polycystic kidney disease (ARPKD). Five (50%) had extrarenal manifestations; retinitis pigmentosa in 3, polydactyly in 1and skeletal dysplasia in 1. Six (60%) had confirmed genetic disease out of which 4 had a pathogenic variant in *SDCCAG8, DYNC2H1, NPHP 1*, and *CEP164* and 2 had likely pathogenic variant in *PKHD1*. Two had VUS in *PKD1* and *PKD2*. In one child (case 1) the clinical diagnosis was reclassified from Bardet Biedl Syndrome to nephronophthisis 15. In Case 32, a new diagnosis of short-rib thoracic dysplasia was made. In one child (case 72) clinical diagnosis of ADPKD (large kidneys with cysts in kidneys and liver) was reclassified to ARPKD. One child (case 10) had no genetic variant despite having a strong phenotype of nephronophthisis realted ciliopathy (ESKD and retinitis pigmentosa with an affected sibling of a similar phenotype).

### Nephrolithiases/nephrocalcinoses

Of 11 children with nephrolithiasis and/or nephrocalcinosis, clinical diagnoses were primary hyperoxaluria (*n* = 8), uric acid stone with hyperuricemia (*n* = 2) and familial hypomagnesemia, hypercalciuria and nephrocalcinosis (FHHNC) (*n* = 1). Nine (81%) had a pathogenic variant. Five had a pathogenic variant in the *AGXT* gene leading to the diagnosis of primary hyperoxaluria type 1. All of them had ESKD at presentation. One child (case 27) who had normal kidney function had a likely pathogenic variant in *HOGA1*. One child had a VUS in the *GRHPR* gene. Two (case 12, 14) with hyperuricemia, AKI and obstructive stones at 2 and 10 months of life were found to have a pathogenic variant in the *HPRT1* gene leading to the diagnosis of Lesch Nyhan syndrome. Apart from hyperuricemia, there were no other features of Lesch Nyhan syndrome in these children. The child with suspected FHHNC had a VUS in *Claudin 16.*

### Tubulopathies

Among 13 children with a clinical diagnosis of tubular disorders, 4 had Fanconi syndrome, 2 suspected distal RTA, 2 bartter syndrome, 2 rickets, 1 dent disease and 2 unknown tubulopathies. Seven (53.8%) had pathogenic variant; 1 *SLC12A1, SLC4A1, SLCA2, FAH1, PHEX, CTNS* and *ATP6V0A4.* No causal variants were identified in 3 children with unclear clinical diagnoses.

### CKD with unknown etiologies

CES was done in four children with CKD with unknown etiology before transplant. One girl (case 35) had a Pathogenic variant in *COL4A4* leading to the diagnosis of autosomal recessive Alport syndrome. On family screening, the younger sibling was found to harbor the same pathogenic variant leading to early diagnosis of Alport’s syndrome. One boy (case 44) had a homozygous pathogenic variant in *COL4A5* while one child (case 64) had a homozygous VUS in the *BBS4* gene. The other two children had no genetic variant.

## Discussion

The study presents two points that are of interest**.** First, the feasibility of genetic testing in a clinical setting using a combination of methods for sequencing and second the impact of genetic diagnosis in the management of kidney disease and family screening in an unselected cohort. In the study, the genetic cause for kidney disease was identified in 35 out of 76 children (46%). The solve rate was high in children with nephrolithiasis and/or nephrocalcinosis (8/11;81%), ciliopathies/cystic kidney diseases (6/10; 60%), tubular disorders (7/13; 53.8%) and was least in glomerular diseases (10 of 34;29.4%). With the help of genetic testing, in 50% of children (2/4) with CKD with unknown etiology, a specific cause could be ascertained. Children with a pathogenic variant were younger at disease onset than those without a genetic etiology. This was similar to earlier reports, where the probability of having a pathogenic variant increase with younger age and decreases as age increases [10]. Although a majority of the children (81%) with genetic disease had a homozygous variant with autosomal recessive inheritance, family history was present in only 4. Hence it is important to note that the absence of family history should not be a factor in not suspecting a genetic cause [11].

The high yield in our cohort substantiates the use of genetic testing in establishing a molecular diagnosis. Low median age, 10% consanguinity and detailed phenotyping in the cohort before sending genetic test were probably predictive of the high diagnostic yield. In published studies, the diagnostic yield of genetic testing varies from 6.3 to 100% depending on the characteristics of the cohort and the method of analysis employed [3]. In a cohort of 127 patients ranging from newborn to 81 years, the overall solve rate of massively parallel sequencing by a kidney disease panel (KidneySeq v1, 177 genes) was 43% (54 of 127 patients) [10]. The solve rate was 46% in patients between 0–14 years, which decreased to 22% for patients > 30 years old. The solve rate (46%) in our cohort was similar. Though CES was not done in all the children with SRNS, the proportion (28%) of children with a pathogenic variant was similar to the median of 26% reported in earlier cohorts [3, 10, 12–16]. One-third of children had VUS, mostly novel with a phenotypically severe disease similar to those with a monogenic form, indicating a high probability of having a genetic cause.

Whole exome sequencing in patients < 25 years with either nephrolithiasis or nephrocalcinosis detected a monogenic cause in 29% in an earlier study [17]. However, 9 of 11 children (81%) with nephrolithiasis and nephrocalcinosis in the current study had a pathogenic variant, probably reflecting a carefully selected cohort where a genetic test was done. Similarly, the yield of a causal variant in tubulopathies in our cohort was high (53.8%) which was similar to the previous report in European cohorts [18, 19]. Two-thirds of children with cystic kidney disease had a pathogenic variant, which was much higher than reported in one study (12%) but similar to a median of 50% based on two earlier studies [3, 20–22].

CKDu is frequently seen in CKD cohorts; 6% at 12–15 years, 21% at 18–21 years and 36% of all cases with adult-onset CKD do not have a diagnosis [23]. Massively parallel sequencing has been increasingly used in the CKD population and is found to be useful in providing an alternative strategy to obtain a definitive diagnosis as recently demonstrated in 9 out of 92 patients by Lata et al. [24] and in 16 of 34 families (47%) in a cohort of 114 Irish families [25]. Two out of 4 children with CKDu (50%) had a pathogenic variant in our study. It established the diagnosis of autosomal recessive Alport syndrome and X linked Alport syndrome in these children.

In 20 out of 35 children with a genetic diagnosis, genetic testing correlated with the phenotype thereby confirming the diagnosis and further helped in prognostication, clinical management, and genetic counselling. For example, diagnosis of primary hyperoxaluria was confirmed in 6 children. Renal prognosis is different in different types of primary hyperoxaluria. Hence, genetic testing in hyperoxaluria not only helps establish the diagnosis but also to know the specific type of hyperoxaluria. In addition, it informs about the approach to transplant as Type I hyperoxaluria requires combined kidney-liver transplantation while in Type II, only kidney transplant would suffice.

In 15 children (19.7%) genetic testing provided a new diagnosis or revised the initial diagnosis. A correct diagnosis by genetic testing helps in counselling, facilitates living donor selection, and clarifies the risk of recurrence post-renal transplantation. For example, detection of a pathogenic variant in the *NPHP1* gene established the diagnosis of nephronophthisis in a child suspected of FSGS progressed to ESKD (case 5). It not only unequivocally established the cause of kidney failure but also clarified disease recurrence risk post kidney transplantation. In case 72, the diagnosis was changed from ADPKD to ARPKD after the detection of a pathogenic variant in the *PKHD1* gene. Reclassification or establishing a new diagnosis helps in reverse phenotyping in children. For example, in two children with CKD (case 36, 44) in whom etiology was unknown, diagnosis of Alport syndrome was made based on detection of homozygous pathogenic variant in *COL4A4* and *COL4A5* genes. This has important management implications as these children require screening for deafness as well as evaluation of the eyes. Similarly, detection of a heterozygous pathogenic variant in intron 9 of *WT1* lead to the diagnosis of Frasier syndrome in a girl (case 67), who was then found to have 46 XY in karyotyping, complete sex reversal and gonadal dysgenesis. Further, immunosuppression was stopped as a therapeutic response was unlikely. This is a powerful demonstration of personalized medicine based on a genetic diagnosis.

Massively parallel sequencing has increasingly helped in identifying phenocopy. A child (case 70) with dense medullary nephrocalcinosis and suspected primary hyperoxaluria based on a high urine oxalate excretion, was diagnosed to have Bartter syndrome type 2 after detection of a homozygous pathogenic variant in the *KCNJ 1* gene. It has important therapeutic as well as prognostic implications. The child did not have typical symptoms of Bartter syndrome. Repeat investigation following genetic results did reveal metabolic alkalosis and high urinary chloride establishing the correct diagnosis and helped in initiating appropriate treatment.

Diagnosis of genetic disease helps in the detection of disease in other family members as well as for antenatal counselling. For example, case36 was diagnosed with ARAS (pathogenic variant in *COL4A4*) when CES was done to evaluate the cause of CKD. Sanger sequencing of the same gene in her younger sibling who had microscopic hematuria, led to the discovery of the same variant (case 76). Detection of the same genetic variant in *HPRT1* and *AGXT* gene in two fetuses as observed in index cases with Lesch nehan syndrome and hyperoxaluria respectively helped in appropriate counselling and termination of pregnancy.

While massively parallel sequencing based testing has many utilities, there are limitations too. Despite advances in bioinformatics, pathogenicity in some cases will remain uncertain. Twenty-four children (31.5%) had VUS, in whom definitive pathogenicity could not be established although phenotypically they were strongly suspected of having a genetic disease. Identification of VUS (mostly novel variants) as observed in a significant proportion of children in the current study poses challenges in interpretation and conveying the information to parents. In addition, segregation studies in the family to ascertain causality adds to the cost. A girl (case 11) with a heterozygous VUS in the *PLCE1* gene developed recurrence of FSGS post kidney transplantation proving that the variant identified in the child was not causal. Hence, critical assessment of reported variants must occur before clinical decision-making is influenced by genetic findings. Besides VUS, 17 children did not have a genetic variant. Two siblings had a phenotype of Senior Loken syndrome and ESKD but no pathogenic variant was detected in one of the siblings (case 10) who was tested. Inability to detect causal variants could be due to limitations in testing strategies or identification of the variant in a gene that is yet to be associated with the phenotype or when the disease has a complex inheritance pattern (e.g. Digenic variants). Whole-exome sequencing could be useful in these cases as clinical exome sequencing might not detect a novel variant or copy number variations. These limitations and the requirement of additional testing in the family should be informed to parents before ordering genetic testing. In 6 children, a VUS was identified as a secondary variant along with a pathogenic variant. The significance of these variants could not be found out and they remain a clinical challenge. Also, being a retrospective review, detailed clinical information like GFR was lacking in many patients which is a major limitation of this study.

## Conclusion

Genetic testing was useful in confirming a suspected diagnosis, making a new diagnosis, reverse phenotyping, genetic counselling, and personalized treatment in our cohort. Detailed phenotyping probably increased the yield of genetic testing. However, detection of a variant of uncertain significance remains a significant clinical challenge.

## Data Availability

The datasets generated and/or analyzed during the current study are available in the NCBI repository at https://www.ncbi.nlm.nih.gov/bioproject/PRJNA863410
